# Identification of Adequate Neurally Adjusted Ventilatory Assist (NAVA) During Systematic Increases in the NAVA Level

**DOI:** 10.1109/TBME.2011.2159790

**Published:** 2011-06-16

**Authors:** Dimitrios Ververidis, Mark van Gils, Christina Passath, Jukka Takala, Lukas Brander

**Affiliations:** 1 VTT Technical Research Centre of Finland Tampere33101; 2 Department of Intensive Care MedicineBern University Hospital (Inselspital) and University of Bern 3010 Bern Switzerland

**Keywords:** Diaphragm electrical activity, neurally adjusted ventilatory assist, patient-ventilator interaction

## Abstract

Neurally adjusted ventilatory assist (NAVA) delivers airway pressure (P_aw_) in proportion to the electrical activity of the diaphragm (EAdi) using an adjustable proportionality constant (NAVA level, cm⋅H _2_O/$\mu$V). During systematic increases in the NAVA level, feedback-controlled down-regulation of the EAdi results in a characteristic two-phased response in P_aw_ and tidal volume (Vt). The transition from the 1st to the 2nd response phase allows identification of adequate unloading of the respiratory muscles with NAVA (NAVA_AL_). We aimed to develop and validate a mathematical algorithm to identify NAVA_AL_. P_aw_, Vt, and EAdi were recorded while systematically increasing the NAVA level in 19 adult patients. In a multistep approach, inspiratory P_aw_ peaks were first identified by dividing the EAdi into inspiratory portions using Gaussian mixture modeling. Two polynomials were then fitted onto the curves of both P_aw_ peaks and Vt. The beginning of the P_aw_ and Vt plateaus, and thus NAVA _AL_, was identified at the minimum of squared polynomial derivative and polynomial fitting errors. A graphical user interface was developed in the Matlab computing environment. Median NAVA_AL_ visually estimated by 18 independent physicians was 2.7 (range 0.4 to 5.8) cm⋅H _2_O/$\mu$V and identified by our model was 2.6 (range 0.6 to 5.0) cm⋅H _2_O/$\mu$V. NAVA_AL_ identified by our model was below the range of visually estimated NAVA_AL_ in two instances and was above in one instance. We conclude that our model identifies NAVA_AL_ in most instances with acceptable accuracy for application in clinical routine and research.

## I. Introduction

**Fig. 1.**
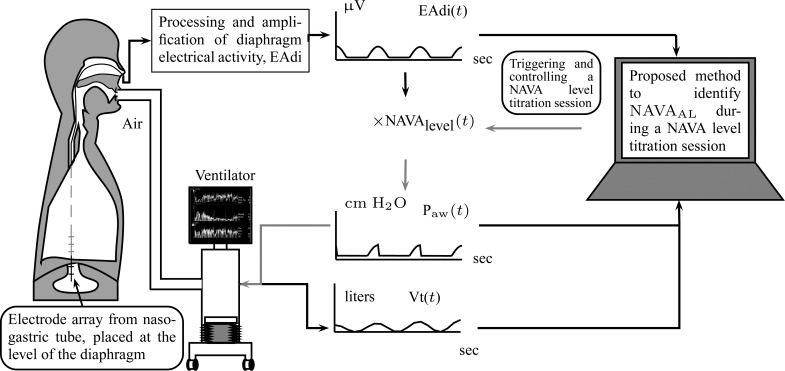
Principles of neurally adjusted ventilatory assist (NAVA) [1]. The diaphragm electrical activity (EAdi) derived from electrodes on a naso-gastric feeding tube is first amplified and processed. The EAdi signal is then multiplied by an adjustable gain factor (NAVA level) and used to control the pressure generator of a mechanical ventilator. Thus, NAVA delivers pressure to the airways (${\rm P}_{{\rm aw}}$) in direct synchrony and linear proportionality to the patient's neural inspiratory drive as reflected by the EAdi (${\rm P}_{{\rm aw}}(t)$ = EAdi($t$) ⋅ ${\rm NAVA}_{{\rm level}}$($t$). Vt = tidal volume. $\hbox{NAVA}_{\rm {AL}}$ = NAVA level that provides adequate unloading of respiratory muscles.

Neurally adjusted ventilatory assist (NAVA) is a new mode of mechanical ventilation that delivers airway pressure (${\rm P}_{{\rm aw}}$) in linear proportion to the electrical activity of the diaphragm (EAdi), a signal arising from the diaphragm's neural activation during spontaneous breathing (Fig. 1) [1]. The NAVA level refers to an adjustable proportionality constant that determines the amount of ${\rm P}_{{\rm aw}}$ delivered per unit of EAdi. Thus, ${\rm P}_{{\rm aw}}(t)$ [cm ⋅H_2_O] = EAdi($t$) [$\mu$V] ⋅ ${\rm NAVA}_{{\rm level}}$($t$) [cm ⋅H_2_O/ $\mu$V]. EAdi is a validated measure of global respiratory drive that is controlled via lung-protective feedback mechanisms, which integrate information from pulmonary and extra-pulmonary mechanoreceptors, from blood gases, and from voluntary input [2]–[5]. If the assist delivered with NAVA exceeds the subject's respiratory demand, EAdi is reflexively down regulated, resulting in less assist for the same NAVA level and vice versa [6]–[11].

Several experimental and clinical studies with NAVA demonstrated that during ramp increases in the NAVA level, transpulmonary pressure and tidal volume (Vt) initially increase (1st response) before being limited due to feedback-controlled down-regulation of EAdi (2nd response) [6][7][8][9]–[11]. Hence, the breathing pattern response to systematic increases in the NAVA level is directed towards prevention of lung overdistension [6], [7], [8], [9], [10], [12]. Interestingly, in rabbits loaded with various inspiratory resistors, the transition from the 1st to the 2nd response phase occurred when the animals' inspiratory effort was reduced to levels similar to those observed during spontaneous breathing (i.e., when breathing without assist and without additional load) [10]. Thus the transition from the 1st to the 2nd response phase presumably reflects the transition from an initial insufficient ventilatory assist to an adequate level of respiratory muscle unloading ($\hbox{NAVA}_{\rm {AL}}$). Therefore, reliable identification of $\hbox{NAVA}_{\rm {AL}}$ during a NAVA level titration procedure is of potential clinical relevance, since it may help to individualize the support level during NAVA.

We hypothesized that identification of $\hbox{NAVA}_{\rm {AL}}$ can be modeled. In Section II, we aimed to develop a mathematical algorithm that would objectively identify the transition from the 1st to the 2nd response phase based on ${\rm P}_{{\rm aw}}$ and Vt responses during NAVA level titration procedures that were performed in a previously reported clinical study on 19 critically ill adults [11]. In Section III, $\hbox{NAVA}_{\rm {AL}}$ as identified by the algorithm was compared to $\hbox{NAVA}_{\rm {AL}}$ as visually estimated by 18 independent observers [11]. A discussion of the method is outlined in Section IV, and conclusions are drawn in Section V.

## II. Development of an Algorithm to Calculate $\hbox{NAVA}_{\rm {AL}}$

**Fig. 2.**
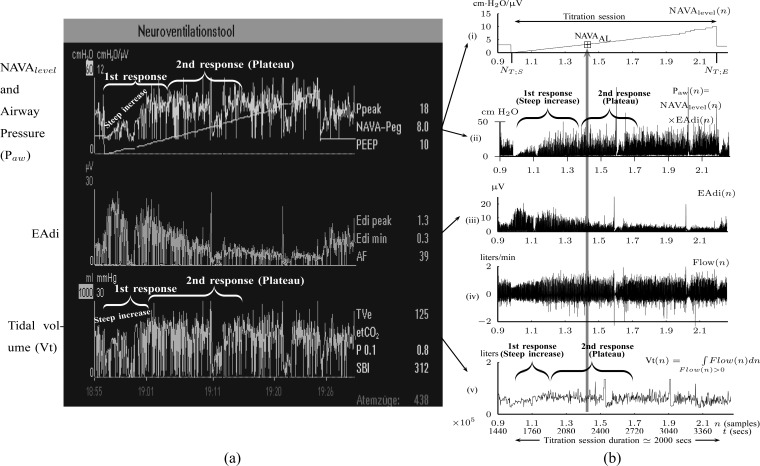
Example of a NAVA level titration session as used for estimating $\hbox{NAVA}_{\rm {AL}}$(a) visually or (b) with the proposed algorithm. $\hbox{NAVA}_{\rm {AL}}$ refers to the adequate NAVA level early after the transition from the initial steep increase in ${\rm P}_{{\rm aw}}(n)$ and Vt($n$), referred to as 1st response, to the less steep increase or plateau in ${\rm P}_{{\rm aw}}(n)$ and Vt($n$), referred to as 2nd response [6]–[11]. Flow($n$) is the air flow. In (a), the Vt($n$) is estimated on a breath-by-breath basis. If there is false triggering of the ventilator (e.g., based on an EAdi artifact) a minimal Vt (normally a few milliliters) is delivered. Since there is no minimal threshold for Vt, the ventilator displays whatever Vt($n$) is delivered in the graph. In (b), the Vt($n$) is calculated as the integral of Flow($n$) per inspiration as it is described in Section II-B (Step 4A).

Identification of $\hbox{NAVA}_{\rm {AL}}$ is based on the analysis of EAdi, ${\rm P}_{{\rm aw}}$, and Vt recordings while systematically increasing the NAVA level. The principles of such a NAVA level titration procedure have been described elsewhere [6], [7], [9]–[11]. Briefly, first the NAVA level was reduced to a minimum of 0 cm⋅H_2_O/$\mu$V. When sufficient EAdi was detectable (i.e., at least twice the EAdi trigger threshold), the NAVA level was increased by 0.1 cm ⋅H_2_O/ $\mu$V every 20 sec while continuously monitoring and recording the EAdi, Paw, and Vt signals (NAVA tracker, Maquet, Solna, Sweden) in NT1 format. The NT1 files were converted into Matlab format for further processing. In the study by Passath et al. [11], the data of one patient were recorded with different software and were, therefore, not included in the experimental part of the present work. A characteristic example of such a titration session is depicted in Fig. 2.

### A. Visual Estimation of $\hbox{NAVA}_{\rm {AL}}$

A visual method for estimating $\hbox{NAVA}_{\rm {AL}}$ was described and validated recently [6], [7], [9]–[11]. Briefly, by observing time plots of ${\rm P}_{{\rm aw}}$ and Vt on the ventilator monitor or on printouts (Fig. 2), $\hbox{NAVA}_{\rm {AL}}$ was determined as the NAVA level early after the transition from an initial steep increase in ${\rm P}_{{\rm aw}}(n)$ and Vt($n$) (1st response) to a less steep increase or even a plateau in both parameters (2nd response). For validation of the visual method, an arbitrarily chosen number of 17 independent physicians blinded to the $\hbox{NAVA}_{\rm {AL}}$ selected during the study were instructed post-hoc identify a NAVA level immediately following the transition from a steep to a less steep increase in ${\rm P}_{{\rm aw}}$ and Vt on screen prints of the original trend graphs. The $\hbox{NAVA}_{\rm {AL}}$ as estimated during the clinical study and post-hoc by the 17 independent physicians was reported previously [11] and used for comparison to $\hbox{NAVA}_{\rm {AL}}$, as identified by the algorithm developed in the present study.

### B. Algorithm-Based Calculation of $\hbox{NAVA}_{\rm {AL}}$

**Fig. 3.**
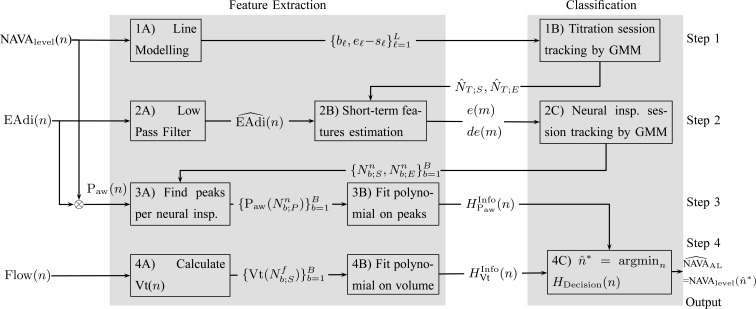
Outline of the algorithm to identify $\hbox{NAVA}_{\rm {AL}}$ based on the signals ${\rm NAVA}_{{\rm level}}$($n$) for the NAVA level, ${\rm EAdi}(n)$ for electrical activity of the diaphragm, and Vt$(n)$ for tidal volume that was derived from the inspiratory flow.

The method to mathematically identify $\hbox{NAVA}_{\rm {AL}}$ is divided into four steps. The procedure is outlined in Fig. 3. The first step is the identification of the titration session from ${\rm NAVA}_{{\rm level}}$($n$) represented by nodes 1(A) and 1(B). The second step is the tracking of inspiration sessions from ${\rm EAdi}(n)$ represented by nodes 2(A), 2(B), and 2(C). The third step consists of identifying the peaks in the ${\rm P}_{{\rm aw}}(n)$ per inspiration and of fitting a polynomial function to the ${\rm P}_{{\rm aw}}$ peaks, as shown in nodes 3(A) and 3(B), respectively. The fourth step consists of calculating Vt($n$) from Flow($n$), and fitting a polynomial function to the Vt, as shown in nodes 4(A) and 4(B). The derivation of $\hbox{NAVA}_{\rm {AL}}$ based on polynomials can be found in node 4(C). The sampling rate of all signals used was $F_s = 62.5$ Hz. All steps are described in greater detail below.

#### 1. Step 1. Identification of the titration session based on changes in the ${\rm NAVA}_{{\rm level}}$$(n)$

*1A*) Let $N_{T;S}$ and $N_{T;E}$ denote the samples where titration session starts and ends, respectively. We wish to identify $N_{T;S}$ and $N_{T;E}$. ${\rm NAVA}_{{\rm level}}$($n$) is modeled with $L$ straight line segments as $\{{\cal L}_{\ell }\}_{\ell =1}^L = \{(a_{\ell }, b_{\ell }, s_{\ell }, e_{\ell })\}_{\ell =1}^{L}$ where

$${\rm NAVA}_{\rm level}(n) = a_{\ell } n + b_{\ell }\ \hbox{for}\ n = \{s_{\ell }, s_{\ell }+1, \ldots, e_{\ell }\} \eqno{\hbox{(1)}}$$

with $\ell$ being the index of the line segment ${\cal L}_{\ell }$, $a_{\ell }$ the first-order line coefficient, $b_{\ell }$ the zero-order coefficient, $s_{\ell }$ the starting sample, and $e_{\ell }$ the ending sample of the $\ell$th line segment. It should be noted that there is no noise in ${\rm NAVA}_{{\rm level}}$($n$). The line segments are found by fitting a sequence of lines to ${\rm NAVA}_{{\rm level}}$*n*) as follows. The first line is fitted to ${\rm NAVA}_{{\rm level}}$($n$) for $s_1=1$ to $e_1=2$. $e_1$ is updated by $e_1=e_1+1$ as long as

$${\rm NAVA}_{\rm level}(e_1+1) = a_{\ell }(e_1+1) + b_{\ell }. \eqno{\hbox{(2)}}$$

**Fig. 4.**
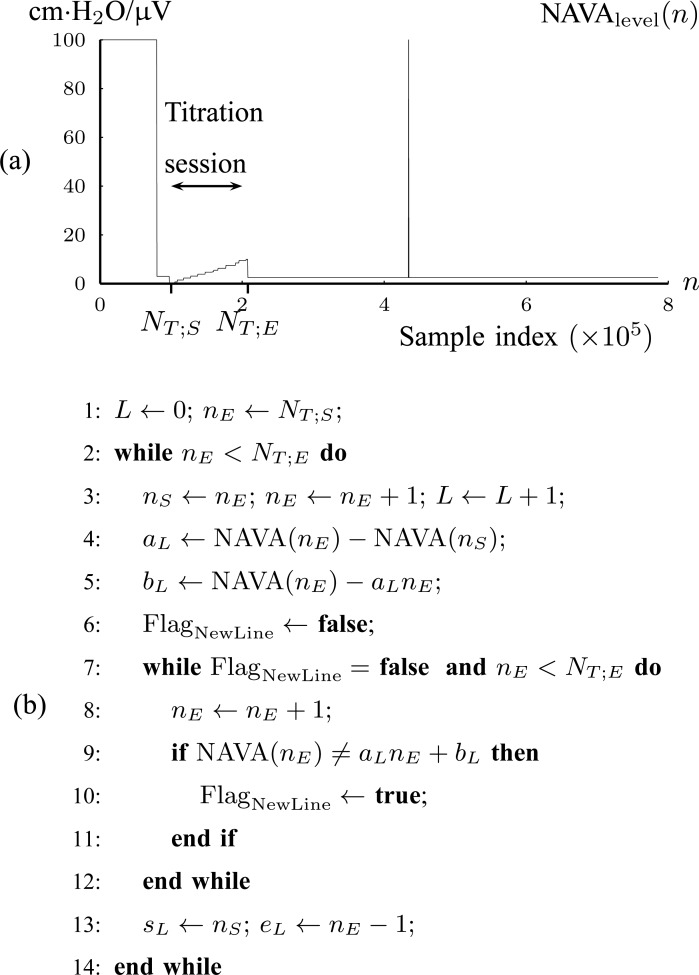
(a) Tracking of the NAVA level titration session in Patient 1 (Step 1). (b) Algorithm for modeling {${\rm NAVA}_{{\rm level}}$($n)\}_{n=1}^N$ with lines $\{{\cal L}_{\ell }\}_{\ell }^L=\{(a_{\ell }, b_{\ell }, s_{\ell }, e_{\ell })\}_{\ell =1}^{L}$ (Step 1A).

If (2) is violated, a new line begins, estimated from the next two samples. The benefit of this transformation of ${\rm NAVA}_{{\rm level}}$($n$) into lines is that a great compression of signal data is accomplished. The algorithm is summarized in Fig. 4(b).

**Fig. 5.**
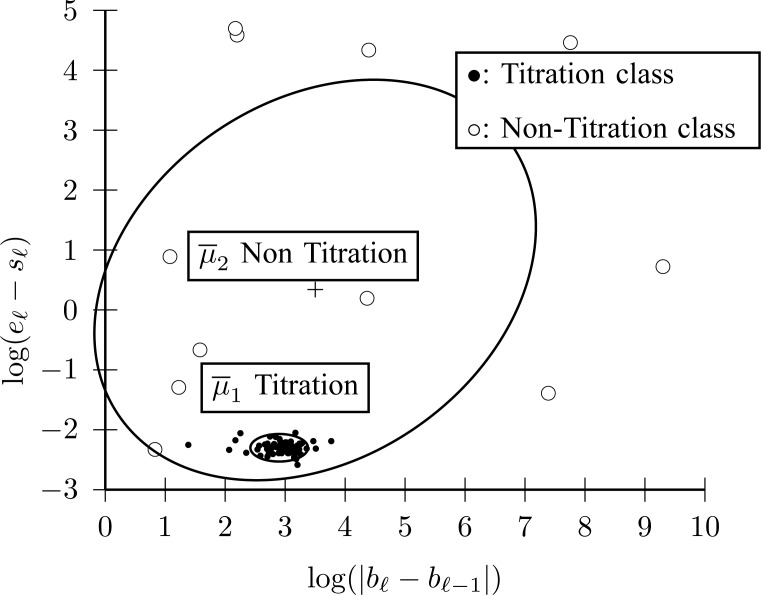
${\rm NAVA}_{{\rm level}}$($n$) titration session tracking by 2 Gaussian components for Fig. 4. The component with small dispersion corresponds to Titration class (Step 1B).

1B) Let $\overline{x}_{\ell } = [\log (\vert b_{\ell }-b_{\ell -1}\vert) \;\; \log (e_{\ell }-s_{\ell })]$ be the 2-D vector that will be used for classifying ${\cal L}_{\ell }$ into $\Omega_1$ (Titration class) or into $\Omega_2$ (Nontitration class). The first feature of $\overline{x}_{\ell }$ is the difference of offset level between the previous and current line segments, which, according to the inspection of Fig. 4(a), should be an almost constant number for ${\cal L}_{\ell } \in \Omega_1$. The second feature of $\overline{x}_{\ell }$ is the length of each line, which should also be a statistically constant number for ${\cal L}_{\ell } \in \Omega_1$. A Gaussian Mixture Modelling (GMM) algorithm is used that searches for a component with a small determinant in $\{\overline{x}_{\ell }\}_{\ell }^L$ space where the number of components is limited to 2. The algorithm used for GMM was found in a previous investigation and is publicly available [13], [14]. Let ${\cal G}(\overline{\mu },\Sigma)$ denote a Gaussian component, with $\overline{\mu }$ and $\Sigma$ being its mean vector and its covariance matrix, respectively. Thus, ${\cal G}(\overline{\mu }_1,\Sigma_1)$ and ${\cal G}(\overline{\mu }_2,\Sigma_2)$ are found, where $\Vert \Sigma_1\Vert < \Vert \Sigma_2\Vert$, with $\Vert \cdot \Vert$ being the determinant of a matrix inside the delimiters. The titration tracking procedure of the signal of Fig. 4(a) is depicted in Fig. 5. A prediction $\hat{c}_{\ell }$ for each line is given according to the Bayes classifier

$$\hat{c}_{\ell } = \mathop{\rm {argmax}}\limits_{c=1,2} P(\overline{x}_{\ell }\vert \Omega_c) \eqno{\hbox{(3)}}$$

where the probability density function (pdf) for each class is given by $P(\overline{x}_{\ell }\vert \Omega_c) = {\cal M}{\cal V}{\cal N}(\overline{x}_{\ell }\vert \overline{\mu }_c,\Sigma_c)$, with ${\cal M}{\cal V}{\cal N}(\overline{x}_i; \overline{\mu }, \overline{\Sigma })$ being the multivariate normal pdf. Let $\hat{N}_{T;S}$ and $\hat{N}_{T;E}$ be the estimated sample index where titration starts and ends, respectively. Then $\hat{N}_{T;S}=s_{\ell_1}$ and $\hat{N}_{T;E}=e_{\ell_2}$, where

$$\eqalignno{\ell_1 &:= \mathop{\rm {argmin}}\limits_{\ell }(s_{\ell }\vert {\cal L}_{\ell } \in \Omega_1) &{\hbox{(4)}}\cr \ell_2 &:= \mathop{\rm {argmax}}\limits_{\ell }(e_{\ell }\vert {\cal L}_{\ell } \in \Omega_1) &\hbox{(5)}}$$

**Fig. 6.**
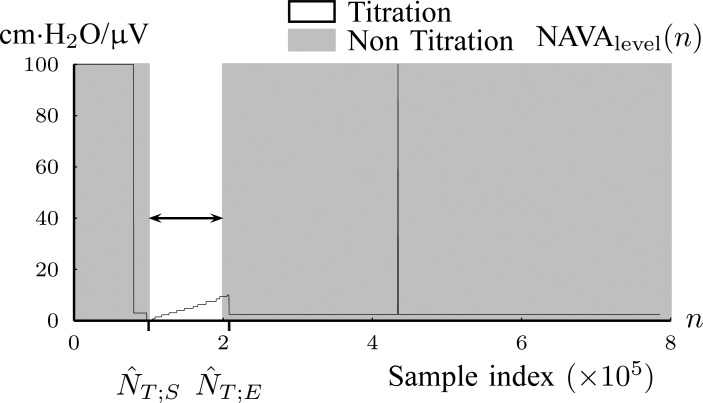
The result of titration tracking procedure of Fig. 5. The lines that belong to ${\cal G}(\overline{\mu }_1,\Sigma_1)$ are assigned to the Titration class (Step 1B).

The estimated $[\hat{N}_{T;S},\hat{N}_{T;E}]$ interval is depicted in Fig. 6. The benefit of this step is that the titration session is tracked without the need of a trigger input from the ventilation machine.

#### 2. Step 2. Tracking of neural inspiration sessions

The electrical activity of the diaphragm, denoted as ${\rm EAdi}(n)$ for $n=1,2,\ldots, N$ is used to track neural inspiration sessions. This is accomplished by employing the GMM clustering algorithm that searches for three Gaussian components in 2-D feature space. The first feature is the logarithm of the short-term energy, estimated as follows.

2A) A moving average (low pass filter, LPF) of order 40 is applied to ${\rm EAdi}(n)$ to eliminate frequency components above 4 Hz that are not related to breathing, i.e.,

$$\widehat{\rm EAdi}(n) = {1\over 40} \sum_{i=0}^{39} {\rm EAdi}(n-i). \eqno{\hbox{(6)}}$$

**Fig. 7.**
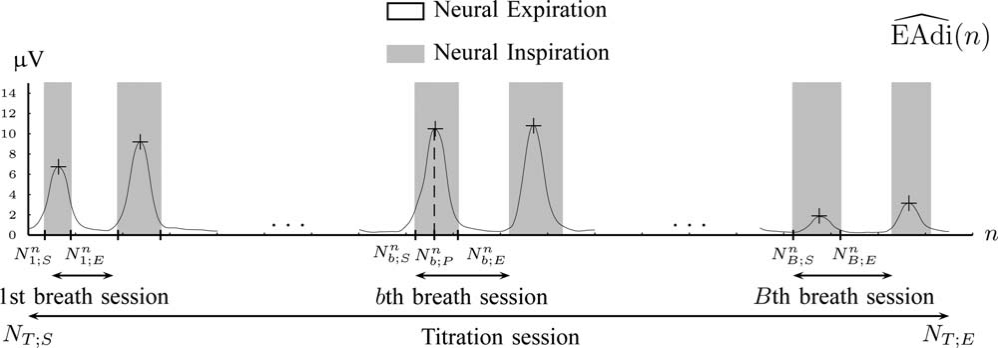
Tracking of neural inspiration sessions using $\widehat{{\rm EAdi}}(n)$ signal (Step 2C).

The $\widehat{{\rm EAdi}}(n)$ for Patient 1 is shown in Fig. 7, where only 6 breaths out of 350 are shown for visualization reasons. The LPF does not introduce negative values of $\widehat{{\rm EAdi}}(n)$ that cause problems when the logarithm operator is applied in the following step.

2B) Next, short-term energy is estimated. That is, $\widehat{{\rm EAdi}}(n)$ is split into frames $f_{\widehat{{\rm EAdi}}}(n;m) = \widehat{{\rm EAdi}}(n)\cdot w(m-n)$, where $w(m-n)$ is an orthogonal window of length $N_w$ ending at sample $m$. In our investigation $N_w$ equals 15, and $m$ starts from 15 samples, which correspond to 240 msec. m is updated by m:=m+15. Patients in intensive care typically have breath cycles of approximately 1 to 4 sec duration. Overlapping is avoided because each sample should be assigned to one class. The first feature is the logarithm of energy for the $\widehat{{\rm EAdi}}(n)$ frame ending at $m$

$$e(i)=\log \Big {(}{1\over N_w} \sum_{n=m-N_w+1}^{m} [f_{\widehat{\rm EAdi}}(n;m)]^2\Big {)} \eqno{\hbox{(7)}}$$

where $i=1,2,\ldots, N/m$. The second feature is the derivative of the first feature, given by $de(i) = e(i) - e(i-1)$. The energy and the energy derivative are chosen because the $\widehat{{\rm EAdi}}(n)$ curve should be divided into valleys (expirations) and mountains (inspirations). It was found experimentally that the logarithm operator transforms the distribution of energy from exponential to normal. In this manner, the GMM clustering algorithm can be applied to the feature distribution as described next.

**Fig. 8.**
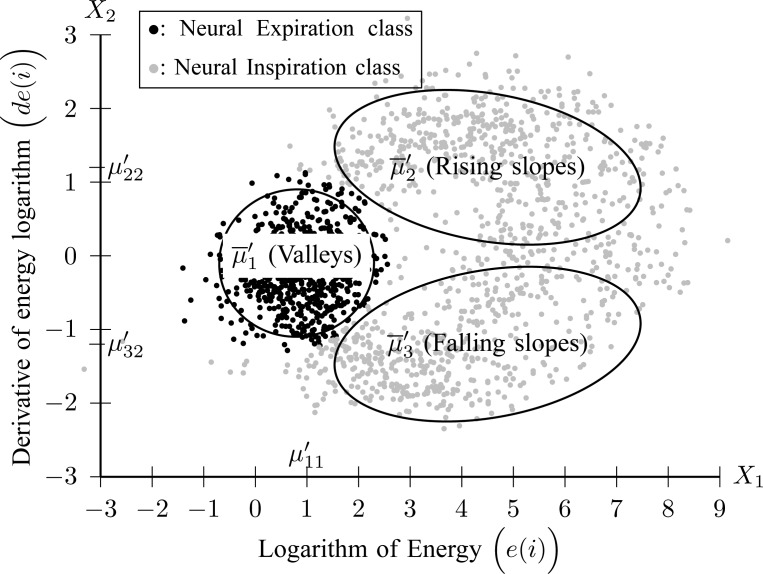
Clustering of $\widehat{{\rm EAdi}}(n)$ frames to Neural Inspiration and Expiration classes (Step 2C).

2C) GMM is applied to feature space $\overline{x}_i=[e(i)\; de(i)]$ where three Gaussian components are searched for. The clustering result for Patient 1 is depicted in Fig. 8.

Each component ${\cal G}(\overline{\mu }^{\prime }_j, \Sigma^{\prime }_j)$ is described by its center ($\overline{\mu }^{\prime }_j = [\mu^{\prime }_{j1}\;\mu^{\prime }_{j2}]$) and its covariance matrix ($\Sigma^{\prime }_j$), for $j=1,2,3$. The component with the center of lowest energy $\mu^{\prime }_{11}$ corresponds to Neural Expiration class, denoted as $\Omega^{\prime }_1$. The Neural Inspiration class, denoted as $\Omega^{\prime }_2$, consists of two Gaussian components. The component with a center signified by maximum derivative of energy $\mu^{\prime }_{21}$ corresponds to rising slopes, and the component signified by minimum derivative of energy $\mu^{\prime }_{23}$ stands for falling slopes of $\widehat{{\rm EAdi}}(n)$. The Bayes classifier is again employed in order to assign each frame to Inspiration or Expiration class. Let $u_i$ be a frame with measurements $\overline{x}_i$ and label $c_i$. The predicted label of $u_i$ is given by $\hat{c}_i = \mathop{\hbox{argmax}}_{c=1,2} P(\overline{x}_i\vert \Omega^{\prime }_c)$, with $P(\overline{x}_i\vert \Omega^{\prime }_1) = {\cal M}{\cal V}{\cal N}(\overline{x}_i; \overline{\mu }^{\prime }_1, \Sigma^{\prime }_1)$ and $P(\overline{x}_i\vert \Omega^{\prime }_2)={\cal M}{\cal V}{\cal N}(\overline{x}_i; \overline{\mu }^{\prime }_2, \Sigma^{\prime }_2) + {\cal M}{\cal V}{\cal N}(\overline{x}_i; \overline{\mu }^{\prime }_3, \Sigma^{\prime }_3)$.

A neural inspiration session is constituted by a sequence of frames that belong to the Neural Inspiration class ($\Omega^{\prime }_2$). The results of this step are shown in Fig. 7. Let $b=1,2,\ldots, B$ be the breath index, where $B$ is the total number of breaths. The beginning and the end of the $b$th neural inspiration session are denoted as $N^n_{b;S}$ and $N^n_{b;E}$, respectively.

*3A) Neural inspiration peaks estimation:* Let ${\rm P}_{{\rm aw}}(n)=\hbox{NAVA}_{{\rm level}}(n)\cdot {\rm EAdi}(n)$ be the airway pressure signal. The neural inspiration peaks indices are found by

$$N^n_{b;P} = \mathop{\rm {argmax}}\limits_{N^n_{b;S}}^{N^n_{b;E}} {\rm P}_{\rm aw}(n) \eqno{\hbox{(8)}}$$

for $b=1,2,\ldots, B$. The airway pressure at neural inspiration peaks is the signal $\{{\rm P}_{{\rm aw}}(N^n_{b;P})\}_{b=1}^B$.

*3B) Polynomial fit to airway pressure peaks:* The polynomial

$${H_{\rm P}}_{\rm aw}(n) = \sum_{k=1}^{K} q_k n^k \eqno{\hbox{(9)}}$$

of order $K=10$, with $q_k$ being the polynomial coefficients, is fitted onto $\{{\rm P}_{{\rm aw}}(N^n_{b;P})\}_{b=1}^B$ with the reweighted least-squares method [15]. By finding the $\mathop{\hbox{argmin}}_n \Big {[}{dH_{{\rm P}_{{\rm aw}}}(n)\over dn} \Big {]}^2$ one is able to derive the time index of plateau of airway pressure peaks. The order of the polynomial is chosen empirically, so that it is a trade-off between tracking the underlying number of curve peaks and capturing the trivial sudden peaks. However, this is not the only information needed for choosing the optimum time index. Also, the signal formed by the sequence of polynomial fit error values

$${\varepsilon_{\rm P}}_{\rm aw}(N^n_{b;P}) = \sqrt{{\vert H_{\rm P}}_{\rm aw}(N^n_{b;P})- {\rm P}_{\rm aw}(N^n_{b;P})\vert} \eqno{\hbox{(10)}}$$

for $b=1,2,\ldots, B$ is taken into consideration. ${\rm P}_{{\rm aw}}(n)$ peaks may present great variance around the fitted polynomial, a fact denoting the patient's inability to synchronize his breath with the ventilation machine. So, another polynomial of order $K-1$ is fitted onto $\varepsilon_{{\rm P}_{{\rm aw}}}(N^n_{b;P})$, i.e.,

$${{{H_\varepsilon}_{\rm P}}_{\rm aw}}(n) = \sum_{k=1}^{K-1} q^{\varepsilon }_k n^k \eqno{\hbox{(11)}}$$

with $q^{\varepsilon }_k$ being its coefficients. The polynomial of $2K-2$ order

$$H^{\rm Info}_{{\rm P}_{\rm aw}}(n) = \Big {[}{dH_{{\rm P}_{\rm aw}} (n)\over dn} \Big {]}^2 + [H_{\varepsilon_{{\rm P}_{\rm aw}}}(n)]^2 \eqno{\hbox{(12)}}$$

includes both information about airway pressure peaks plateau and small variance, where the latter indicates that the plateau is stable.

**Fig. 9.**
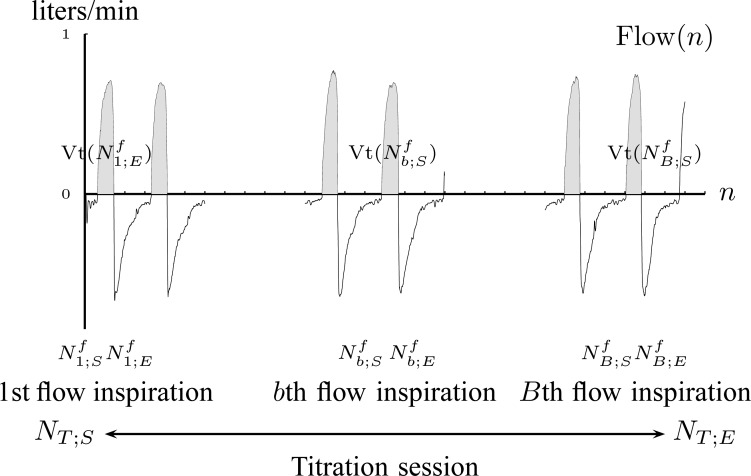
The air flow signal, ${\rm Flow}(n)$, is divided into inspirations and expirations by zero crossing indices (Step 4A).

*4A) Tidal volume estimation:* The ${\rm Flow}(n)$ signal for Patient 1 is depicted in Fig. 9.

Let the tidal volume ${\rm Vt}(N^f_{b;S})$ be the air inhaled during $b$th flow inspiration, where $N^f_{b;S}$ and $N^f_{b;E}$ are the starting and ending index of $b$th airflow inspiration. A flow inspiration session is defined as the time during which air flow is positive. So, a flow inspiration session is found by applying the zero crossings method on ${\rm Flow}(n)$. Then, the tidal volume is found by integrating the inspiration flow for each $b=1,2,\ldots, B$ inspiration

$${\rm Vt}(N^f_{b;S}) = {1\over F_s} \sum_{n=N^f_{b;S}}^{N^f_{b;E}} {\rm Flow}(n). \eqno{\hbox{(13)}}$$

*4B) Polynomial fit to tidal volume:* The polynomial

$$H_{\rm{Vt}}(n) = \sum_{k=1}^{K} r_k n^k \eqno{\hbox{(14)}}$$

is fitted onto $\{{\rm Vt}(N^f_{b;S})\}_{b=1}^B$, where $r_k$ are the polynomial coefficients, in a similar manner as in Step 3B. The sequence of fit errors, i.e.,

$$\varepsilon_{\rm{Vt}}(N^f_{b;S}) = \sqrt{\vert H_{\rm{Vt}}(N^f_{b;S})- \rm{Vt}(N^f_{b;S})\vert } \eqno{\hbox{(15)}}$$

for $b=1,2,\ldots, B$ is also exploited. The polynomial

$$H_{\varepsilon_{\rm{Vt}}}(n) = \sum_{k=1}^{K-1} r^{\varepsilon }_k n^k \eqno{\hbox{(16)}}$$

is fitted onto (15), where $r^{\varepsilon }_k$ are the polynomial coefficients. So, the information about the tidal volume plateau and its variance is given by

$$H^{\rm{Info}}_{\rm{Vt}}(n) = \Big {[}{dH_{\rm{Vt}}(n)\over dn} \Big {]}^2 + [H_{\varepsilon_{\rm{Vt}}}(n)]^2. \eqno{\hbox{(17)}}$$

**Fig. 10.**
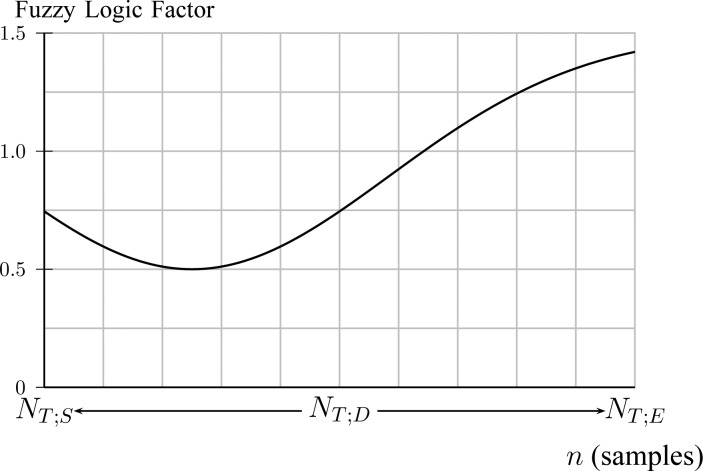
A fuzzy logic factor used for exploiting $n^*$ bias to 0.25 of total duration of titration session (Step 4C).

*4C) Estimation of plateau:* $\hbox{NAVA}_{\rm {AL}}$ equals a certain ${\rm NAVA}_{{\rm level}}$($n$) when signals $\{{\rm P}_{{\rm aw}}(N^n_{b;P})\}_{b=1}^B$ and $\{{\rm Vt}(N^f_{b;S})\}_{b=1}^B$ reach a plateau and simultaneously present small variance around the fitted polynomial. Let $n^*$ be the time index when the plateau occurs and small variance is observed. An estimate of $n^*$, denoted as $\hat{n}^*$ is found when both (12) and (17) are minimized. A function that includes information about the time index where polynomial derivatives and fitting errors are minimized is

$$\eqalignno{&H_{\rm Decision}(n) = \Big {[}H^{{\rm Info}_{\rm P}}_{\rm aw}(n) + H^{\rm Info}_{\rm Vt}(n)\Big {]} \cdot \cr &\underbrace{\Big {(}1.5 - {{\cal M}{\cal V}{\cal N}(n; {N_{T;D}\over 4},{N_{T;D}\over 3})\over {\rm max} {{\cal M}{\cal V}{\cal N}(n; {N_{T;D}\over 4},{N_{T;D}\over 3})}} \Big {)}}_{\hbox{Fuzzy logic factor}} &\hbox{(18)}}$$

where the fuzzy logic factor is plotted in Fig. 10.

The fuzzy logic factor is biased toward the first quarter of titration session duration. It will be shown in experiments that physicians are highly biased at $\hbox{NAVA}_{\rm {AL}}$ = 2.5. Since NAVA is increasing from 0 to 10 linearly through time, this corresponds to a bias in time toward $0.25 N_{T;D}$. The optimum time index is then given by

$$\hat{n}^* = \mathop{\rm {argmin}}\limits_{{n=N_{T;S}}}^{N_{T;E}} H_{\rm Decision}(n). \eqno{\hbox{(19)}}$$

**Fig. 11.**
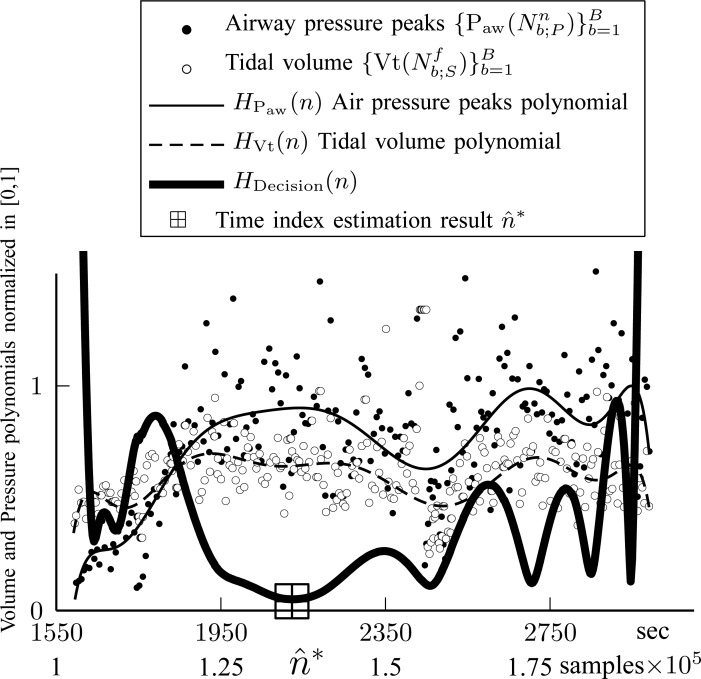
Time index of plateau, $\hat{n}^*$, is found when $H_{{\rm Decision}}(n)$ is minimized, as described in Steps 3 and 4.

Finally, we define $\widehat{\hbox{NAVA}}_{\rm {AL}}=\hbox{NAVA}_{{\rm level}}(\hat{n}^*)$. As an example, in Fig. 11, the curves resulting from (9), (14), and (18) are plotted for Patient 1.

The signals $\{{\rm P}_{{\rm aw}}(N^n_{b;P})\}_{b=1}^B$ and $\{{\rm Vt}(N^f_{b;S})\}_{b=1}^B$ are also plotted in order to demonstrate the polynomial fitting. It is inferred that $H_{{\rm Decision}}(n)$ is minimized at $\hat{n}^*= 2114$, which is close to $n^*= 2065$ which was given by the clinician. The $\hbox{NAVA}_{\rm {AL}}$ is 2.5, whereas the algorithm found $\widehat{\hbox{NAVA}}_{\rm {AL}}=2.7$.

## III. Experiments

For all titration sessions performed in the 19 patients, $\hbox{NAVA}_{\rm {AL}}$ calculated by our algorithm was compared to $\hbox{NAVA}_{\rm {AL}}$ as visually estimated by the investigators when performing the clinical study (i.e., by author LB) and by an arbitrarily chosen number of 17 independent physician observers posthoc using printouts of the signal trajectories [Fig. 2(a)] [11]. Median $\hbox{NAVA}_{\rm {AL}}$, as estimated by the 18 physicians, was 2.5 cm⋅H_2_O/$\mu$V with a range from 0.4 to 5.8 cm ⋅H_2_O/ $\mu$V. In the study by Passath et al. [11], the number of steps necessary to reach $\hbox{NAVA}_{\rm {AL}}$ and the highest NAVA level used differed among patients. The highest NAVA level used in the 19 patients included in the present work was (median [range]) 4.9 (1.9–7.4) cm⋅H_2_O/$\mu$V and the time to reach this level was 978 (377–1478) sec. The time to reach $\hbox{NAVA}_{\rm {AL}}$ was 498 (198–997) sec.

**Fig. 12.**
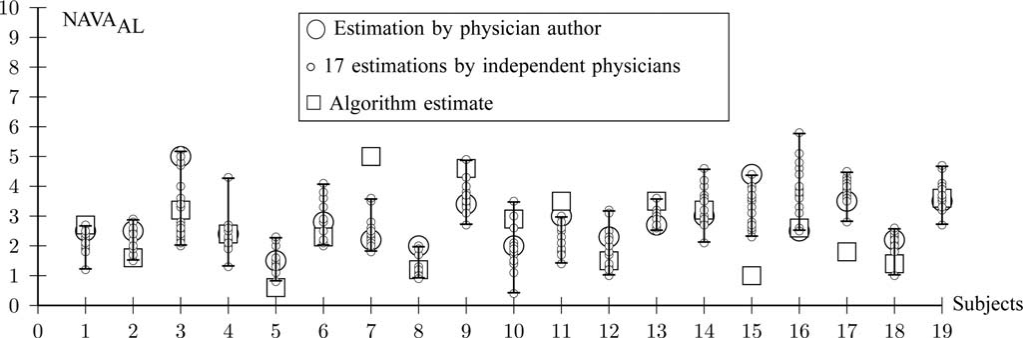
Comparison between $\hbox{NAVA}_{\rm {AL}}$ independently estimated by one of the authors (L.B., a physician) and by 17 independent physicians based on visual inspection of the airway pressure (${\rm P}_{{\rm aw}}$) and tidal volume (Vt) response to systematic increases in the NAVA level (circles) and $\hbox{NAVA}_{\rm {AL}}$ identified by the algorithm described in this paper (squares).

Median $\hbox{NAVA}_{\rm {AL}}$ identified by the algorithm was 2.6 cm⋅H_2_O/$\mu$V with a range from 0.6 to 5.0 cm ⋅H_2_O/ $\mu$V. In most cases, $\hbox{NAVA}_{\rm {AL}}$ identified by the algorithm was within the range of $\hbox{NAVA}_{\rm {AL}}$ estimated by the physicians (Fig. 12). In Patient 7, the $\hbox{NAVA}_{\rm {AL}}$ identified by the algorithm was higher, and in Patients 15 and 17 it was lower than the $\hbox{NAVA}_{\rm {AL}}$ estimated by the physicians. In order to calculate the correlation between $\hbox{NAVA}_{\rm {AL}}$, as identified by the observers with the results of our algorithm, we computed the multiple correlation coefficient (MCC) [16]. MCC ranges from 0 (no correlation) to 1 (linearly dependent). In our case, MCC indicates the correlation between the matrix of $\hbox{NAVA}_{\rm {AL}}$ estimates for all observers across all patients with the algorithm result. Furthermore, the Pearson concordance coefficient is used to estimate the concordance between a single observer and the algorithm [11]. The confidence limits are estimated at 95% level of significance. The MCC between $\hbox{NAVA}_{\rm {AL}}$ as identified by the algorithm and as estimated by the 18 physicians is 0.54±0.06. The Pearson concordance coefficients between the $\hbox{NAVA}_{\rm {AL}}$ as identified by each observer and the algorithm are presented in Table I. In the last row, the concordance between median $\hbox{NAVA}_{\rm {AL}}$ for all observers and the algorithm is computed. It can be seen that the concordance of the $\hbox{NAVA}_{\rm {AL}}$ between each observer and the algorithm is always positive. The lower limit of the concordance coefficient is slightly negative, with a median value of −0.13. The upper confidence limit median is 0.69.

**Table I:** Pearson Concordance Coefficient of $\hbox{NAVA}_{\rm {AL}}$ Estimates between Physician Observers and Algorithm

Observer	Coefficient	Lower Limit	Upper limit
1 (author L.B.)	0.21	−0.27	0.61
2	0.25	−0.23	0.63
3	0.41	−0.06	0.73
4	0.37	−0.10	0.71
5	0.40	−0.06	0.72
6	0.52	0.09	0.79
7	0.41	−0.05	0.73
8	0.20	−0.28	0.60
MI 9	0.36	−0.11	0.70
10	0.28	−0.20	**0.65**
11	0.24	−0.24	0.62
12	0.48	0.03	**0.77**
13	0.33	−0.15	0.68
14	0.20	−0.28	0.60
15	0.41	−0.06	0.73
16	0.23	−0.25	0.62
17	0.14	−0.33	0.56
18	0.43	−0.03	0.74
Median observer	0.34	−0.13	0.69

**Fig. 13.**
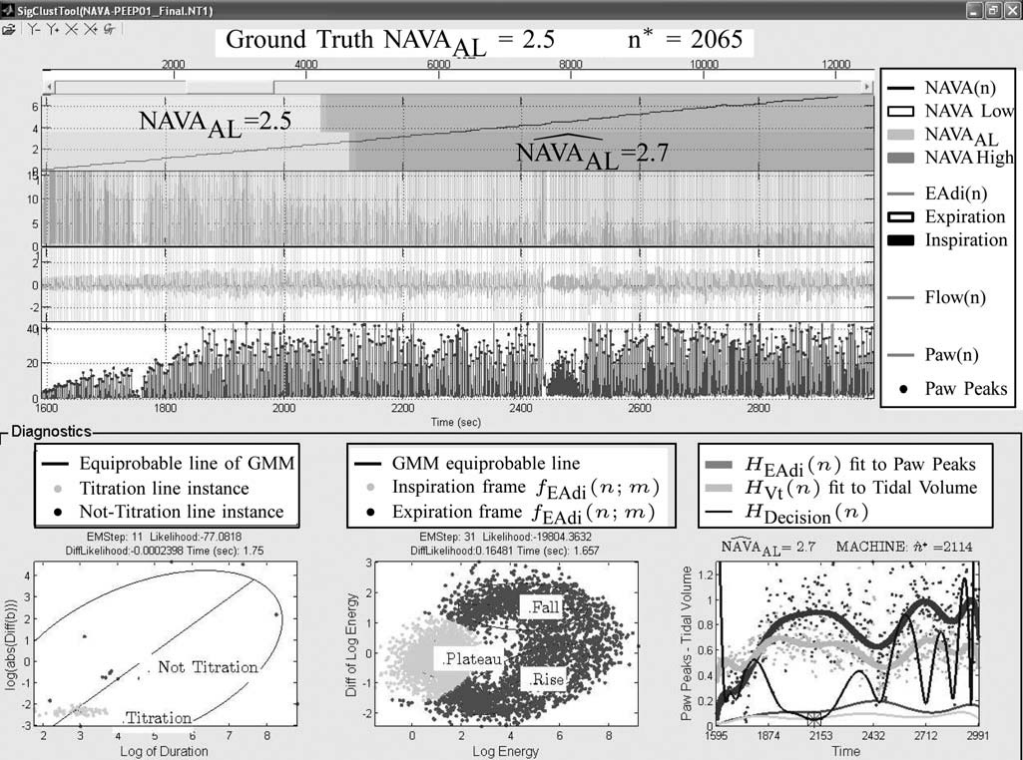
The graphic interface provides a synopsis of the signal processing steps described in Figs. 2, 5, 8, and 11, and allows for real time assessment of how the algorithm identifies $\hbox{NAVA}_{\rm {AL}}$. Ground truth $\hbox{NAVA}_{\rm {AL}}$ denotes the visually estimated adequate NAVA level.

A graphic user interface (GUI) for the algorithm is presented in Fig. 13. The GUI includes most of the figures presented in Section II-B. The final result is compared to the ground truth, i.e., the $\hbox{NAVA}_{\rm {AL}}$ estimated visually, and displayed as bands in the uppermost panel of Fig. 13.

## IV. Discussion

We developed a multistep algorithm and a user interface to identify adequate assist ($\hbox{NAVA}_{\rm {AL}}$) based on analysis of the Vt, ${\rm P}_{{\rm aw}}$, and EAdi responses during a systematic increase in the NAVA level. The algorithm revealed results that were comparable to the previously used visual method for estimating $\hbox{NAVA}_{\rm {AL}}$.

Delivering mechanical ventilatory assist during spontaneous breathing aims at unloading the respiratory muscles from excessive work of breathing while preventing both fatigue and disuse atrophy of respiratory muscles. However, determining an assist level that adequately meets the patient's needs is not straightforward. Both too high and too low assist may cause harm. While respiratory muscle fatigue may result from insufficiently unloading the patient from his work of breathing [17], disuse atrophy may follow prolonged delivery of assist in excess of the patient's needs [18]–[20].

Thus, defining an adequate level of respiratory muscle unloading based on the patient's individual response to changes in the assist level is of clinical relevance but requires reliable measurement of the respiratory drive. The recent introduction of a technology to monitor EAdi, a validated measure of respiratory drive [2]–[5], provides the opportunity to integrate the patient's response in the process of identifying an adequate level of assist. NAVA is unique in that it directly translates changes in the respiratory drive into changes of the ventilatory pattern. Since with NAVA the ventilator receives the same control signal as the diaphragm, it conceptually acts as an additional external respiratory muscle pump that is directly controlled by the patient's respiratory drive. Thus, NAVA provides the patient with far-reaching control over the ventilatory pattern and with the ability to limit the assist once the inspiratory efforts occur at a level that corresponds to nonloaded conditions, i.e., at a satisfactory, and hence adequate, assist level with NAVA ($\hbox{NAVA}_{\rm {AL}}$) [6], [7], [9]–[11].

In the present study, we demonstrate that $\hbox{NAVA}_{\rm {AL}}$ can be identified using a multistep polynomial fitting model based on analyzing the Vt, Paw, and EAdi responses during systematic increases in the NAVA level. The $\hbox{NAVA}_{\rm {AL}}$ identified by the algorithm was in agreement with the $\hbox{NAVA}_{\rm {AL}}$ estimated visually for most patients. We previously demonstrated not only good reproducibility among physicians for visual estimation of $\hbox{NAVA}_{\rm {AL}}$ [10], [11] but also stable cardio-pulmonary function without evidence of respiratory failure or distress when implementing $\hbox{NAVA}_{\rm {AL}}$ for various time spans [6], [7], [9]–[11].

**Fig. 14.**
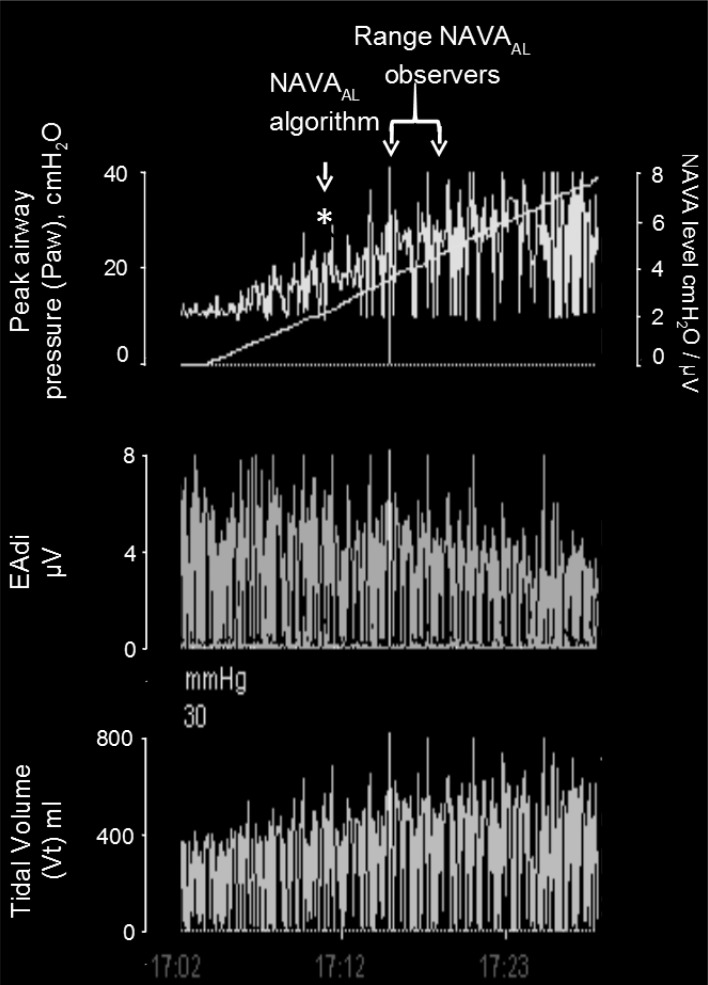
NAVA level titration session in patient 17. In this patient the algorithm identified the transition from a steep increase in peak airway pressure (${\rm P}_{{\rm aw}}$) to a less steep increase or plateau in ${\rm P}_{{\rm aw}}$ (i.e., the adequate NAVA level, $\hbox{NAVA}_{\rm {AL}}$) clearly below the range of $\hbox{NAVA}_{\rm {AL}}$ as visually estimated by the clinicians. The discrepancy is most likely due to a short, transitory interruption of the ${\rm P}_{{\rm aw}}$ increase during the initial steep increase, i.e., during the 1st response phase (asterisk). We assume that the physicians outperformed the current version of the algorithm in recognizing pattern irregularities.

In 3 out of 19 titration sessions, the $\hbox{NAVA}_{\rm {AL}}$ identified by the algorithm was either clearly above or clearly below the range of $\hbox{NAVA}_{\rm {AL}}$ estimated visually. We assume that the discrepancy between the methods in these three patients is most likely due to the fact that the physicians outperformed the current version of the algorithm in recognizing pattern irregularities, as illustrated in Fig. 14. Also, the current version of the ventilator.s graphic interface does not differentiate between real breaths and artifacts when displaying the trend graphs. Therefore the graphs may be difficult to read for users non-experienced with the NAVA level titration procedure. This suggests that, although $\hbox{NAVA}_{\rm {AL}}$ identified by the algorithm was within the range of $\hbox{NAVA}_{\rm {AL}}$ estimated visually for >80% of the titration sessions, a visual verification is advisable before using $\hbox{NAVA}_{\rm {AL}}$ identified by the current version of the algorithm. Further refinement and validation of the algorithm is required before it can be safely implemented in clinical practice.

Of note, since the transition from the 1st to the 2nd response does not occur acutely, some inter-individual variability and discrepancy between methods used in determining $\hbox{NAVA}_{\rm {AL}}$ can be expected. Also, as ${\rm P}_{{\rm aw}}$ and Vt do not or only minimally change after the transition from the 1st to the 2nd response phase, any NAVA level within the 2nd response phase can be expected to have only minor, if any, effects on breathing pattern.

The mathematical algorithm developed is based on post processing of the signals obtained. The algorithm not only allows faster identification of $\hbox{NAVA}_{\rm {AL}}$ than the visual method but is also independent of observer-related biases and inter-individual variability. However, the algorithm should be modified to identify $\hbox{NAVA}_{\rm {AL}}$ in real-time, and thus help shorten the time needed for a titration session.

## V. Conclusion

$\hbox{NAVA}_{\rm {AL}}$ can be identified quickly and reliably using our polynomial fitting model based on the analysis of the ${\rm P}_{{\rm aw}}$, Vt, and EAdi responses to systematic increases in the NAVA level. The correlation between the $\hbox{NAVA}_{\rm {AL}}$ identified by the algorithm and the $\hbox{NAVA}_{\rm {AL}}$ estimated visually suggests that our model has acceptable accuracy for application in clinical routine and research.
